# Multiple Trajectories of Body Mass Index and Waist Circumference and Their Associations with Hypertension and Blood Pressure in Chinese Adults from 1991 to 2018: A Prospective Study

**DOI:** 10.3390/nu15030751

**Published:** 2023-02-02

**Authors:** Qi Wang, Xiaoyun Song, Shufa Du, Wenwen Du, Chang Su, Jiguo Zhang, Xiaofan Zhang, Xiaofang Jia, Yifei Ouyang, Li Li, Bing Zhang, Huijun Wang

**Affiliations:** 1 Key Laboratory of Trace Element Nutrition of National Health Commission of China, National Institute for Nutrition and Health, Chinese Center for Disease Control and Prevention, Beijing 100050, China; 2 Department of Food, School Hygiene, Dalian Centre for Disease Control and Prevention, Dalian 116035, China; 3 Department of Nutrition and Carolina Population Center, University of North Carolina at Chapel Hill, Chapel Hill, NC 27599, USA

**Keywords:** BMI, WC, multi-trajectory, blood pressure, hypertension, Chinese adults, prospective study

## Abstract

Body mass index (BMI) and waist circumference (WC) have been suggested to be involved in the etiology of hypertension. The present study aimed to determine multiple trajectories of BMI and WC, then examined their associations with the risks of hypertension and high blood pressure in Chinese adults. The study used China Health and Nutrition Survey data from 1991 to 2018. The sample included 9651 adults aged 18 years or older. We used group-based multi-trajectory modeling to identify trajectories. We estimated the relationships between the trajectories and the risks of hypertension with a Cox proportional hazards regression model and the trajectories’ relationships with blood pressure levels with a generalized linear model. We identified four trajectories for each gender: low stable BMI, low increasing WC (group 1); medium increasing BMI, medium increasing WC (group 2); increasing BMI to overweight, increasing WC to central obesity (group 3), increasing BMI to obesity, increasing central obesity WC (group 4). Group 1 was the reference group. Among males in groups 2, 3, and 4, the adjusted hazard ratios (HR) and 95% confidence intervals (95% CI) of hypertension were 1.30 (1.15–1.48), 1.86 (1.58–2.18), and 2.60 (2.02–3.34), respectively. The systolic blood pressure (SBP) and diastolic blood pressure (DBP) of males in group 4 increased by 11.90 mm of mercury (mmHg) and 7.75 mmHg, respectively. Among females in groups 2, 3, and 4, the HR and 95% CI of hypertension were 1.35 (1.18–1.54), 1.92 (1.62–2.26), and 2.37 (1.85–3.03), respectively. The SBP and DBP of females in group 4 increased by 8.84 mmHg and 5.79 mmHg, respectively. These data indicated that increases in BMI and WC were associated with unfavorable hypertension risks. Attention to both BMI and WC trajectories has the potential to prevent hypertension.

## 1. Introduction

Hypertension is a severe public health problem [1]. It is a major cause of premature death worldwide and significantly increases the risks of heart, brain, kidney, and other diseases [2]. In 2021, the World Health Organization estimated that 1.28 billion adults worldwide had hypertension [2]. The prevalence of hypertension among Chinese adults was 27.5% in 2015–2019 [3]. The prevalence of hypertension has increased rapidly over decades [4,5], becoming a heavy burden on the Chinese health care system [6,7]. Therefore, it is critical to identify and control hypertension risk factors to reduce its public health burden.

Obesity is considered an important risk factor for incident hypertension [8,9], and it can be evaluated by many indicators, including body mass index (BMI) and waist circumference (WC) [10,11]. Generally, obesity is divided into general obesity and central obesity [12]. General obesity is mainly defined by BMI, while central obesity is often defined by WC [8,13]. Previous research has shown that BMI and WC were associated with hypertension in both cross-sectional [10,12,14,15,16,17] and longitudinal studies [18,19,20,21]. Trajectories of BMI and WC allow the assessment of dynamic patterns of BMI and WC and the impact on hypertension [22]. Based on the findings of previous studies on BMI trajectories in adults, trajectories of rapidly increasing BMI in Chinese [23] and trajectories of maintaining high BMI levels in Chinese [24] and in Canadians [25] are associated with an increased risk of hypertension. Cheng et al. [26] found that sharply increasing trajectories of WC during adolescence were associated with higher risks of incident hypertension in Chinese. Noushin Sadat Ahanchi et al. [27] found that both increased WC and highly stable trajectories were associated with a higher risk of incident hypertension in Tehran. However, those studies mainly focused on BMI and WC separately, whereas changes in BMI or WC do not occur in isolation [28] but rather simultaneously over time. Recognition of the potential value of considering BMI and WC simultaneously to better understand the risks of hypertension is increasing. To date, the literature on the combined BMI and WC trajectories over time and their association with hypertension remains sparse. We used data from the China Health and Nutrition Survey (CHNS) from 1991 to 2018 to identify multiple trajectories of BMI and WC to examine the associations between these trajectories and the risks of hypertension and blood pressure in Chinses adults. We hypothesized that different trajectory groups may be identified in the population: there may be trajectory groups where both BMI and WC increase or trajectory groups where BMI increases and WC remains stable, and there may be trajectory groups where both BMI and WC remain high. Individuals in different BMI and WC trajectory groups may present different risks of hypertension and different levels of blood pressure. Individuals whose BMI and WC increase rapidly and remain at high levels may have a higher risk of hypertension and higher blood pressure levels.

## 2. Materials and Methods

### 2.1. Study Design

The CHNS is an ongoing population-based longitudinal survey in China that began in 1989. The purpose of this study is to provide evidence for studying the impact of long-term social and economic changes in China on a wide range of nutritional status and health-related outcomes by collecting data from individuals, families, and communities at multiple levels. The CHNS carried out follow-up surveys in 1991, 1993, 1997, 2000, 2004, 2006, 2009, 2011, 2015, and 2018 to collect dietary, anthropometric, clinical, and other individual-, household-, and community-level data. The time interval between each two surveys is 2 years, 4 years, 3 years, 4 years, 2 years, 3 years, 4 years, and 3 years. By 2018, the CHNS sample included people of all ages in 15 Chinese provinces or municipal cities. More details regarding the CHNS are in previous articles [29,30]. Our study included 10 waves of CHNS data: 1991, 1993, 1997, 2000, 2004, 2006, 2009, 2011, 2015, and 2018. The Institutional Review Board of the University of North Carolina at Chapel Hill, Chapel Hill, North Carolina, United States (No. 07-1963), and the Institutional Review Committee of the National Institute for Nutrition and Health, Chinese Center for Disease Control and Prevention, Beijing, China, approved the survey protocols and instruments and the process for obtaining informed consent (No. 201524). All participants provided written informed consent prior to the surveys.

### 2.2. Study Participants

**Figure 1:**
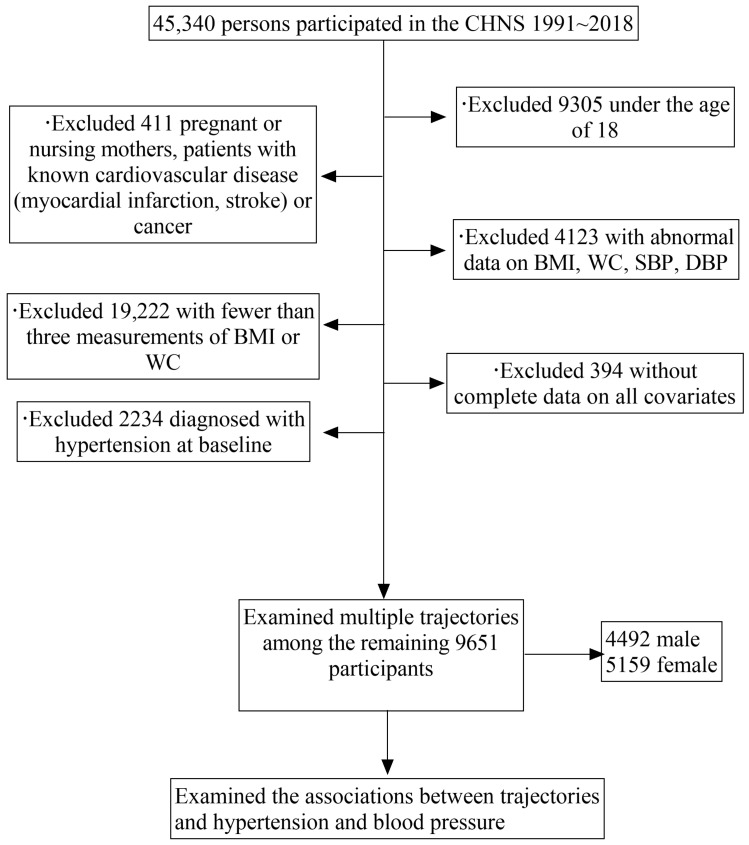
Flowchart of the participants included in the current analysis.

The CHNS included 45,340 participants between 1991 and 2018. We excluded 9305 participants who were younger than 18 years; 411 participants who were pregnant or nursing mothers or patients with known cardiovascular disease (myocardial infarction, stroke) or cancer; 4123 participants with abnormal data on BMI, WC, systolic blood pressure (SBP), or diastolic blood pressure (DBP); 19,222 participants with fewer than three measurements of BMI or WC; 394 participants without complete data on all covariates; and 2234 participants diagnosed with hypertension at baseline (Figure 1). Our study included 9651 participants and 49,139 observations. The number (n) of visits ranged from three to nine per participant: three visits, n of participants = 2824; four visits, n = 1633; five visits, n = 1393; six visits, n = 1285; seven visits, n = 1155; eight visits, n = 874; nine visits, n = 487 (mean five visits).

### 2.3. Measurement of Variables

Trained health workers or nurses collected data on weight, height, WC, SBP, and DBP through physical measurements. Trained investigators measured height without shoes to the nearest 0.1 cm (cm) using a portable SECA stadiometer (SECA, Hamburg, Germany), and they measured body weight without shoes and with light clothing to the nearest 0.1 kg (kg) using a calibrated beam scale (SECA882) by 2015 and using a body composition tester (TANITA BC601) in 2015 and 2018. We calculated BMI as weight in kg divided by height in meters (m) squared (kg/m^2^). We defined overweight as BMI 24.0–27.9 kg/m^2^ and obesity as BMI ≥ 28.0 kg/m^2^ according to the Chinese adult weight criteria (WS/T 428-2013) [31].

Trained investigators measured WC with light clothing to the nearest 0.1 cm using a Seca201 nonelastic tape measure above the participant’s navel while the subject was breathing naturally and standing upright. Central obesity was defined as WC ≥ 90 cm in male and ≥ 85 cm in female according to the Chinese adult weight criteria (WS/T 428-2013) [31].

Investigators measured SBP and DBP on the right arm following standardized procedures using regularly calibrated mercury sphygmomanometers with cuffs. They recorded SBP at the first appearance of a pulse sound (Korotkoff Phase 1) and DBP at the disappearance of the pulse sound (Korotkoff Phase 5). They averaged three measurements of SBP and DBP to reduce the effect of measurement error, and we used the average of the three measurements in our analysis. According to the 2018 Chinese guidelines for the management of hypertension [32], we defined hypertension as SBP ≥ 140 mm of mercury (mmHg) and/or DBP ≥ 90 mmHg, taking antihypertensive medication currently, or a self-reported diagnosis of hypertension.

### 2.4. Assessment of Covariates

Well-trained investigators used standard questionnaires to collect participants’ data. We considered the following measure covariates: age, education level, geographic region (urban or rural), annual per capita household income, survey year, follow-up duration, physical activity, current smoking status, current alcohol drinking status, sodium (Na) intake, and potassium (K) intake. Additional covariates included baseline BMI, WC, SBP, and DBP. The survey year is the time when the participants entered the survey. The follow-up time was the total time the person participated in the survey from the first survey to the last. We used baseline covariates in the study.

Investigators used questionnaires to collect respondents’ recalls of physical activity in four domains: occupation, household, leisure time, and transportation activities. They collected detailed dietary information at the household and individual levels using a weighing method in combination with three consecutive 24 h recalls (1 weekend day and 2 weekdays). We calculated Na intake (milligrams per day [mg/d]) and K intake (mg/d) based on dietary data in the China Food Composition Table.

### 2.5. Statistical Analysis

We summarized characteristics using the median (interquartile range [IQR]) or mean (standard deviation [SD]) for continuous variables and the count (proportion) for discrete variables. We used group-based multi-trajectory modeling to identify multiple trajectories of BMI and WC in both males and females. We performed multi-trajectory modeling with a STATA plug-in using continuous norming distribution for continuous data [33]. We used follow-up years as a timescale for the trajectories. We initiated a model with two trajectory groups and then added three, four, and up to six trajectory groups. Within each given number of trajectory groups, we tested the polynomial orders (cubic, quadratic, linear specifications) for each trajectory shape until we established the best-fitting model. We chose the final number of trajectories based on rigorous criteria. (1) We regarded the model with the lowest Bayesian information criterion (BIC) as a best-fit model. (2) We further tested model adequacy by examining the logged Bayes factor, which is approximately equal to 2∆BIC. Above 10 is recommended. (3) We ascertained within each group the values of average posterior probability of assignment of membership in which values greater than 0.7 indicate adequate internal reliability. (4) We ascertained within each group the values of odds of correct classification. Above 5 for each group is recommended [34].

We used a Cox proportional hazards regression model analysis to examine the associations between the trajectories and the risk of hypertension. Time at entry was the participant’s age at baseline, and exit time was the age at which the participant was diagnosed with hypertension or was lost to follow-up or at which the follow-up period ended, whichever came first. Survival time is the interval between the exit time and the entry time. We calculated hazard ratios (HR) and 95% confidence intervals (95% CI).

We excluded participants who took antihypertensive medication during the study period because medication might have affected their blood pressure values. After testing the normality of residuals and homoscedasticity, we used a generalized linear model to evaluate the associations between BMI and WC trajectory groups and blood pressure. We used data from the participant’s last survey as the outcome.

We fitted five models for both generalized linear model analysis and Cox proportional hazards regression model analysis. We adjusted model 1 for no covariates. We adjusted model 2 for age, education level, geographic region (urban or rural), annual per capita household income, survey year, and follow-up time. We adjusted model 3 additionally for physical activity, current smoking status, current alcohol drinking status, Na intake, and K intake. We further adjusted model 4 for BMI and WC at baseline. We further adjusted model 5 for SBP and DBP at baseline.

For all analyses, we used SAS 9.4 (SAS Institute, Inc., Cary, NC, USA) and Stata SE15 (Stata Corp., College Station, TX, USA). We considered *p* < 0.05 statistically significant.

## 3. Results

### 3.1. BMI and WC Trajectories

**Figure 2:**
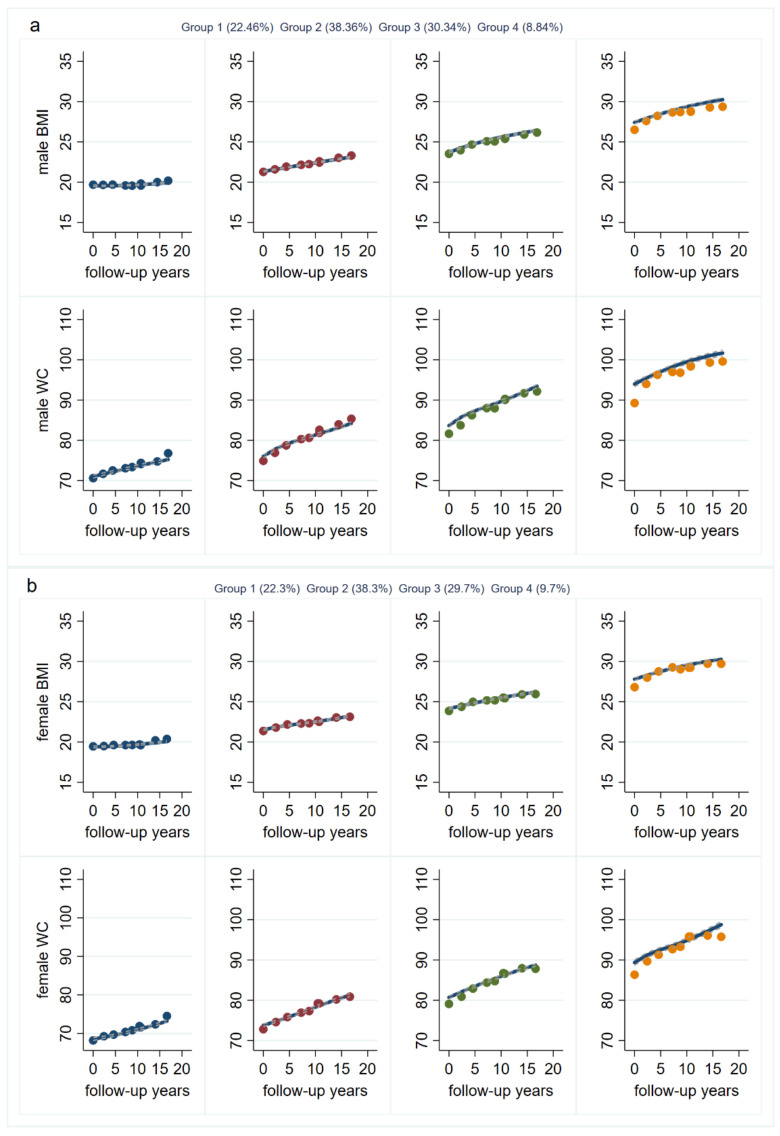
Trajectories of BMI and WC among males (**a**) and females (**b**).

Our sample from 10 waves of CHNS data included 4492 males and 5159 females. According to model-adequacy criteria, the goal of parsimony, and the rule of interpretability, we identified four distinct BMI and WC trajectories in both males and females (Figure 2). Table S1 in the Supplemental Materials presents the parameters of model-adequacy criteria.

Among males, group 1, low stable BMI, low increasing WC, included 22.46% of the male participants. This group’s estimated BMI was around 20 kg/m^2^ and remained stable during the follow-up period. Estimated WC was originally 70 cm and increased to around 75 cm over time. Group 2, medium increasing BMI, medium increasing WC, was the most prevalent with 38.36% of the male participants. Their BMIs ranged from 21 kg/m^2^ to 24 kg/m^2^, increasing slightly over time but notably remaining within the normal range and not approaching overweight. This group’s WCs ranged from 75 cm to 85 cm and increased over time but remained within the normal realm. Group 3, increasing BMI to overweight, increasing WC to central obesity, included 30.34% of the male participants. This group’s estimated BMI increased approximately from 24 kg/m^2^ to 26 kg/m^2^, entering the range of overweight. Group 3’s estimated WC increased from 81 cm to 92 cm, reaching central obesity. Group 4, increasing BMI to obesity, increasing central obesity WC, included the smallest percentage of male participants at 8.84% and had the highest BMIs and WCs at baseline. This group’s BMI at baseline was approximately 27 kg/m^2^ and increased to over 30 kg/m^2^ during the follow-up period to reach obesity. At baseline, group 4’s WC was about 92 cm, a central obesity value, and it increased to over 100 cm.

Among females, group 1, low stable BMI, low increasing WC, comprised 21.92% of the female participants. This group’s BMI at baseline was around 20 kg/m^2^ and increased slightly. The group’s WC increased from 69 cm to 74 cm. As among the males, group 2, medium increasing BMI, medium increasing WC, was the most prevalent with 38.77% of the female participants. This group’s BMIs ranged from 21 kg/m^2^ to 24 kg/m^2^ and increased slightly over time, remaining within the normal level, and not approaching overweight. Group 2’s WCs were between 71 cm and 81 cm and increased over time but remained within the normal level. Group 3, increasing BMI to overweight, increasing WC to central obesity, included 29.77% of the female participants. This group’s BMI increased approximately from 24 kg/m^2^ to 26 kg/m^2^, becoming overweight. Group 3’s WC increased from 80 cm to 90 cm, reaching the level of central obesity. Group 4, increasing BMI to obesity, increasing central obesity WC, included 9.54% of the female participants and had the highest BMIs and WCs at baseline. This group’s baseline BMI was approximately 27 kg/m^2^ and increased to over 30 kg/m^2^ during the follow-up period to reach obesity. At baseline, group 4’s WC was about 90 cm, a central obesity value, and it increased to around 100 cm.

Figure S1 shows the mean BMI and WC of each group at different follow-up times. We found that group-based multi-trajectory modeling do effectively identify BMI and WC trajectory groups.

### 3.2. Baseline Characteristics by Trajectory Group

Our analysis included 9651 individuals, 4492 males and 5159 females. Table 1 details the characteristics of the persons in each trajectory.

The majority of males were in group 2 (38.36%). Among males, group 1 had the lowest education level, the highest proportion of rural residents, the lowest proportion of current drinkers, and the lowest baseline mean levels of BMI, WC, SBP, and DBP compared with other trajectory groups.

The majority of females were in group 2 (38.77%). Among females, group 1 had the lowest baseline mean age, the highest proportion of rural residents, and the lowest baseline mean levels of BMI, WC, SBP, and DBP compared with other trajectory groups.

### 3.3. Associations between Multi-Trajectories and Hypertension

Of the 4492 male participants, 2340 (52.09%) were diagnosed with hypertension during the follow-up. Of these, 145 (6.20%) were diagnosed by self-report, and 95 (4.06%) were diagnosed by taking antihypertensive medication. Table 2 presents the findings of the Cox proportional hazards regression model analyses exploring associations between multiple trajectories and risks of hypertension. Among males, group 1 was the reference group, and group 2 (HR: 1.30, 95% CI: 1.15–1.48), group 3 (HR: 1.86, 95% CI: 1.58–2.18), and group 4 (HR: 2.60, 95% CI: 2.02–3.34) were significantly associated with increased risks of hypertension when adjusted for all covariates of our study.

Of the 5159 female participants, 2330 (45.16%) were diagnosed with hypertension during the follow-up. Of these, 190 (8.15%) were diagnosed by self-report, and 117 (5.02%) were diagnosed by taking antihypertensive medication. Among females, group 1 was the reference group, and group 2 (HR: 1.35, 95% CI: 1.18–1.54), group 3 (HR: 1.92, 95% CI: 1.62–2.26), and group 4 (HR: 2.37, 95% CI: 1.85–3.03) were significantly associated with increased risks of hypertension after adjusting for all covariates of our study. The HRs and 95% CIs of the covariates are in Table S2. Among males, education level, follow-up time, alcohol drinking status, SBP, and DBP all affected hypertension. Among females, education level, WC, SBP, and DBP all affected hypertension. Figure S2 in the Supplemental Materials presents the Kaplan–Meier Curve for model 1 by groups for males and females. We can observe the survival times of the four groups in the figure as a complement to the Cox proportional hazards regression model.

### 3.4. Associations between Multiple Trajectories and SBP and DBP

**Figure 3:**
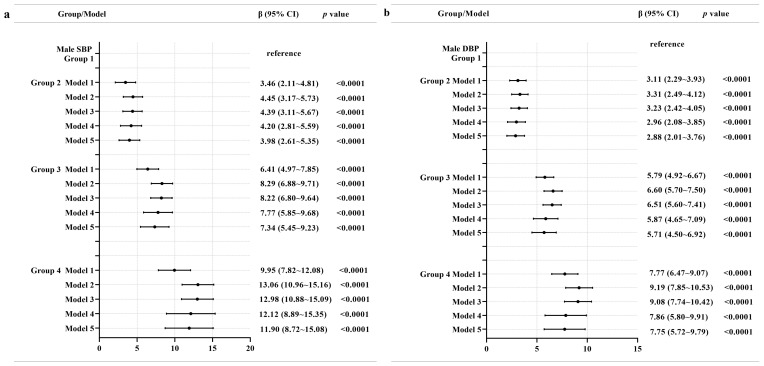
Associations between multiple trajectories and SBP (**a**) and DBP (**b**) in males. Note: Model 1 adjusted for no covariates. Model 2 adjusted for age, education level, geographic region (urban or rural), annual per capita household income, survey year, and follow-up time. Model 3 additionally adjusted for physical activity, current smoking status, current alcohol drinking status, Na intake, and K intake. Model 4 additionally adjusted for BMI and WC at baseline. Model 5 additionally adjusted for SBP and DBP at baseline.

Figure 3 shows the findings of the generalized linear model analyses exploring associations between multiple trajectories and the levels of SBP and DBP among males. Group 2 had 3.98 mmHg higher SBP compared to group 1 on average after adjusting all covariates of our study. Group 3 had 7.34 mmHg higher SBP compared to group 1 on average after adjusting all covariates of our study. Group 4 had 11.90 mmHg higher SBP compared to group 1 on average after adjusting all covariates of our study. In addition, group 2 had 2.88 mmHg higher DBP compared to group 1 on average after adjusting all covariates of our study. Group 3 had 5.71 mmHg higher DBP compared to group 1 on average after adjusting all covariates of our study. Group 4 had 7.75 mmHg higher DBP compared to group 1 on average after adjusting all covariates of our study.

**Figure 4:**
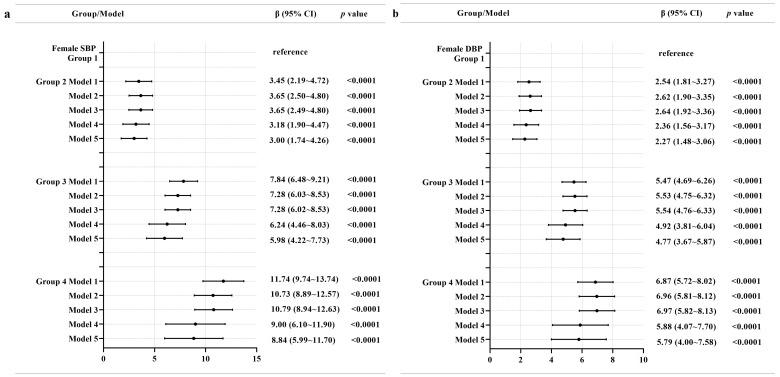
Associations between multiple trajectories and SBP (**a**) and DBP (**b**) in females. Note: Model 1 adjusted for no covariates. Model 2 adjusted for age, education level, geographic region (urban or rural), annual per capita household income, survey year, and follow-up time. Model 3 additionally adjusted for physical activity, current smoking status, current alcohol drinking status, Na intake, and K intake. Model 4 additionally adjusted for BMI and WC at baseline. Model 5 additionally adjusted for SBP and DBP at baseline.

Figure 4 shows that, among females, group 2 had 3.00 mmHg higher SBP compared to group 1 on average after adjusting all covariates of our study. Group 3 had 5.98 mmHg higher SBP compared to group 1 on average after adjusting all covariates of our study. Group 4 had 8.84 mmHg higher SBP compared to group 1 on average after adjusting all covariates of our study. In addition, group 2 had 2.27 mmHg higher DBP compared to group 1 on average after adjusting all covariates of our study. Group 3 had 4.77 mmHg higher DBP compared to group 1 on average after adjusting all covariates of our study. Group 4 had 5.79 mmHg higher DBP compared to group 1 on average after adjusting all covariates of our study.

## 4. Discussion

In this nationwide cohort of Chinese adults, we identified trajectories of BMI and WC as a whole and examined their multi-trajectory associations with hypertension and blood pressure using 10 waves (from 1991 to 2018) of data from the CHNS. We identified four distinct trajectories of BMI and WC in both males and females. In both males and females, we found that the trajectory groups characterized by medium increasing BMI, medium increasing WC; increasing BMI to overweight, increasing WC to central obesity; and increasing BMI to obesity, increasing central obesity WC were prospectively associated with higher risks of hypertension when compared with the group characterized by low stable BMI, low increasing WC. Moreover, we found that the trajectory groups characterized by medium increasing BMI, medium increasing WC; increasing BMI to overweight, increasing WC to central obesity; and increasing BMI to obesity, increasing central obesity WC were prospectively associated with higher levels of SBP and DBP when compared with the group characterized by low stable BMI, low increasing WC.

Multi-trajectory modeling identifies latent clusters of individuals following similar trajectories across multiple indicators of an outcome of interest [35]. To date, we have not found any longitudinal study that determines multiple trajectories of both BMI and WC. The trajectories we identified in this study provide new insights into the simultaneous observation of trajectory patterns of BMI and WC. When grouping the long-term trends of BMI and WC, we found that the multiple trajectories were similar in males and females, but we found some differences. Both BMI and WC showed an increasing trajectory over time, which may be related to the dramatic economic, demographic, and social transformation of China during 1991–2018 [30]. In our four multi-trajectory groups the BMI and WC change patterns were similar in males and females, and the proportions of participants in each group were similar among males and females. The difference is that, in all groups, the WC levels of females were lower than those of males in the corresponding groups. We found that, generally, an increase in BMI is also accompanied by an increase in WC. Studies using data from China [23] and Guatemala [36] that explored trajectories of BMIs in adulthood or throughout the life course by gender showed that BMI may have two to four trajectories in males and females. Though the numbers of trajectories and BMI change patterns were different among those studies with different participants, they all discovered a low increasing and a high increasing or medium increasing group, which is in line with our results. Studies on the trajectories of WCs based on different populations [26,27,37,38] discovered a high increasing group in both males and females, which is similar to our results.

Our analysis of CHNS data found that group 2, group 3, and group 4 were associated with higher risks of hypertension in both males and females. This means that, even for group 2, whose BMIs and WCs were within the normal range and did not meet the criteria for overweight or central obesity, the elevated trajectory of BMI and WC increased the risk of hypertension. Group 3, whose BMIs and WCs were normal at baseline, showed that BMI elevated to overweight levels and WC elevated to central obesity levels over time significantly increased the risk of hypertension. Group 4 had the highest risk of hypertension. Studies investigating the associations between multiple trajectories of both BMI and WC and the risks of hypertension are limited. Some previous research [23,25,26,27,36,37,38,39,40] applied data-driven analytic approaches, such as latent class analysis, to identify distinct and unknown BMI [23,25,36,39,40] or WC [26,27,37,38] univariate trajectories in a population and examined the relationship between trajectories and hypertension. However, those studies mainly focused on BMI and WC trajectories separately, ignoring the fact that changes in BMI and WC are not isolated. We considered both BMI and WC, which could provide practical information for hypertension prevention and intervention.

In our study, group 2, group 3, and group 4 were associated with higher SBP and DBP. Group 4 had the highest SBP and DBP levels. Among males in group 4, the SBP and DBP increased by 11.900 mmHg and 7.750 mmHg, respectively. The respective increases among females in group 4 were 8.840 mmHg and 5.790 mmHg. Previous studies investigated the relationship between only one of BMI or WC and blood pressure levels, which were consistent with the results obtained in our study. H. Fan and X. Zhang [41] identified four distinct BMI trajectories in Chinese children: lean stable increase, medium marked increase, heavy marked decrease, and heavy marked increase. They found that the SBPs and DBPs of the individuals in the medium marked increase group increased on average by 4.147 mmHg and 2.977 mmHg, respectively. The SBPs and DBPs of the individuals in the heavy marked increase group increased on average by 11.660 mmHg and 8.368 mmHg, respectively. W. Fu et al. [42] examined the association between WC and blood pressure cross-sectionally. They found that in males, for each SD increases in BMI, SBP increased by 3.79 mmHg, and for each SD increases in WC, SBP increased by 4.04 mmHg. In females, for each SD increases in BMI, SBP increased by 4.59 mmHg, and for each SD increases in WC, SBP increased by 3.41 mmHg.

According to the life course epidemiology theory [43,44,45], exposure at a particular period in the life span may eventually lead to some diseases later in life. In the current study, the increasing BMI and WC trajectory patterns of individuals may accumulate to affect risks of incident hypertension. This suggests the importance of focusing on the trajectories of BMI and WC over time.

The main strengths of the present study are our application of a multi-trajectory modeling approach to identify multiple codependent BMI and WC trajectories in Chinese adults using long-term repeated data; our long follow-up period; our inclusion of many related covariates; and the novelty of our tests for associations of the trajectories with outcomes and performance analyses separately for males and females, taking into account their differences in BMI and WC. However, our study also has several limitations. First, trajectories identified in this study cannot necessarily be generalized to other ethnic populations or across different time spans. Second, although we adjusted for as many covariates as possible, we cannot rule out the possibility of other confounders we were unable to include, such as dietary factors. Third, many other indicators of obesity besides BMI and WC exist. Further studies should consider more indicators, such as body fat percentage and fat-free mass. Finally, data on 24 h BP, ambulatory blood pressure measurement (ABPM), was not yet available in our study. ABPM can provide significant value in blood pressure measurement and hypertension diagnosis. Such data were not yet available for our study.

## 5. Conclusions

In conclusion, using data from 1991 to 2018, we identified four multi-trajectory BMI and WC groups in the Chinese population and examined associated risks of hypertension and high blood pressure. The medium increasing BMI, medium increasing WC group; the increasing BMI to overweight, increasing WC to central obesity group; and the increasing BMI to obesity, increasing central obesity WC group were associated with higher risks of hypertension and high blood pressure in both males and females. BMI reflects the obesity status of the whole body. We used multiple trajectories of BMI and WC to observe the simultaneous changes in BMI and WC, which is useful for understanding hypertension etiology. These results emphasize the importance of paying attention to both BMI and WC trajectories to prevent hypertension. This gives us a basis for preventing or intervening in hypertension by controlling the increase or magnitude of the increase in BMI and WC. Moreover, this provides a basis for the identification of people at high risk for hypertension, and those with increasing BMI or WC trajectories may be at higher risk for hypertension.

## Figures and Tables

**Table 1:** Baseline characteristics of the study population.

Baseline Characteristics	Trajectory Groups				*p*-Value
Male	Group 1	Group 2	Group 3	Group 4	
N (%)	1009 (22.46)	1723 (38.36)	1363 (30.34)	397 (8.84)	
Age, year (mean [SD])	41.31 (14.80)	39.57 (13.01)	40.75 (12.69)	41.21 (13.33)	0.0080
Education level, N (%)					<0.0001
Primary school and below	491 (48.66)	626 (36.33)	380 (27.88)	72 (18.14)	
Middle school	341 (33.80)	646 (37.49)	501 (36.76)	174 (43.83)	
High school and above	177 (17.54)	451 (26.18)	482 (35.36)	151 (38.04)	
Geographic region, N (%)					<0.0001
Rural	835 (82.76)	1258 (73.01)	801 (58.77)	215 (54.16)	
Urban	174 (17.27)	465 (26.99)	562 (41.23)	182 (45.84)	
Smoking, N (%)					0.0018
Nonsmoker	357 (35.38)	618 (35.87)	544 (39.91)	176 (44.33)	
Current smoker	652 (64.62)	1105 (64.13)	819 (60.09)	221 (55.67)	
Alcohol drinking, N (%)					<0.0001
Nondrinker	426 (42.22)	633 (36.74)	471 (34.56)	123 (30.98)	
Current drinker	583 (57.78)	1090 (63.26)	892 (65.44)	274 (69.02)	
Follow-up time, year (median [IQR])	18.00 (11.00–22.00)	16.00 (9.00–21.00)	14.00 (7.00–18.00)	9.00 (7.00–15.00)	<0.0001
BMI, mg/kg (mean [SD])	19.55 (1.57)	21.28 (1.55)	23.69 (1.93)	27.37 (2.36)	<0.0001
WC, cm (mean [SD])	70.73 (5.61)	76.02 (5.95)	83.69 (7.19)	93.88 (8.23)	<0.0001
SBP, mmHg (mean [SD])	111.37 (10.85)	113.82 (10.22)	117.54 (10.20)	120.28 (9.42)	<0.0001
DBP, mmHg (mean [SD])	72.81 (7.71)	74.27 (7.52)	76.52 (7.10)	78.77 (6.21)	<0.0001
Female	Group 1	Group 2	Group 3	Group 4	
N (%)	1131 (21.92)	2000 (38.77)	1536 (29.77)	492 (9.54)	
Age, year (mean [SD])	39.87 (14.49)	40.05 (12.06)	42.67 (11.91)	44.78 (12.10)	<0.0001
Education level, N (%)					0.0054
Primary school and below	591 (52.25)	955 (47.75)	782 (50.91)	264 (53.66)	
Middle school	294 (25.99)	581 (29.05)	423 (27.54)	154 (31.30)	
High school and above	246 (21.75)	464 (23.20)	331 (21.55)	74 (15.04)	
Geographic region, N (%)					<0.0001
Rural	815 (72.06)	1344 (67.20)	975 (63.48)	313 (63.62)	
Urban	316 (27.94)	656 (32.80)	561 (36.52)	179 (36.38)	
Smoking, N (%)					0.0071
Nonsmoker	1071 (94.69)	1937 (96.85)	1486 (96.74)	468 (95.12)	
Current smoker	60 (5.31)	63 (3.15)	50 (3.26)	24 (4.88)	
Alcohol drinking, N (%)					0.0596
Nondrinker	1025 (90.63)	1791 (89.55)	1345 (87.57)	433 (88.01)	
Current drinker	106 (9.37)	209 (10.45)	191 (12.43)	59 (11.99)	
Follow-up time, year (median [IQR])	16.00 (9.00–21.00)	14.00 (9.00–21.00)	14.00 (9.00–21.00)	12.00 (7.00–18.00)	<0.0001
BMI, mg/kg (mean [SD])	19.34 (1.57)	21.49 (1.62)	24.15 (1.98)	27.82 (2.62)	<0.0001
WC, cm (mean [SD])	68.28 (5.79)	73.75 (6.15)	80.65 (7.07)	89.28 (7.85)	<0.0001
SBP, mmHg (mean [SD])	106.89 (11.82)	109.95 (11.61)	113.50 (11.71)	116.89 (11.08)	<0.0001
DBP, mmHg (mean [SD])	70.15 (8.17)	71.98 (8.08)	74.21 (7.88)	76.18 (7.13)	<0.0001

**Table 2:** Associations between multiple trajectories and risks of hypertension by gender.

Gender	Model	Trajectory Groups						
Male		Group 1 (N = 1009)	Group 2 (N = 1723)		Group 3 (N = 1363)		Group 4 (N = 397)	
		HR	HR (95% CI)	*p*	HR (95% CI)	*p*	HR (95% CI)	*p*
	Model 1	1	1.34 (1.19~1.51)	<0.0001	2.23 (1.98~2.51)	<0.0001	3.86 (3.30~4.52)	<0.0001
	Model 2	1	1.45 (1.29~1.63)	<0.0001	2.36 (2.08~2.67)	<0.0001	3.71 (3.14~4.38)	<0.0001
	Model 3	1	1.43 (1.27~1.61)	<0.0001	2.33 (2.05~2.64)	<0.0001	3.65 (3.09~4.31)	<0.0001
	Model 4	1	1.33 (1.18~1.51)	<0.0001	1.97 (1.68~2.30)	<0.0001	2.65 (2.06~3.41)	<0.0001
	Model 5	1	1.30 (1.15~1.48)	<0.0001	1.86 (1.58~2.18)	<0.0001	2.60 (2.02~3.34)	<0.0001
Female		Group 1 (N = 1131)	Group 2 (N = 2000)		Group 3 (N = 1536)		Group 4 (N = 492)	
		HR	HR (95% CI)	*p*	HR (95% CI)	*p*	HR (95% CI)	*p*
	Model 1	1	1.41 (1.24~1.59)	<0.0001	2.39 (2.11~2.70)	<0.0001	3.75 (3.22~4.36)	<0.0001
	Model 2	1	1.47 (1.30~1.67)	<0.0001	2.30 (2.03~2.61)	<0.0001	3.16 (2.71~3.68)	<0.0001
	Model 3	1	1.48 (1.30~1.67)	<0.0001	2.31 (2.04~2.62)	<0.0001	3.19 (2.73~3.72)	<0.0001
	Model 4	1	1.38 (1.21~1.58)	<0.0001	2.01 (1.70~2.37)	<0.0001	2.51 (1.96~3.21)	<0.0001
	Model 5	1	1.35 (1.18~1.54)	<0.0001	1.92 (1.62~2.26)	<0.0001	2.37 (1.85~3.03)	<0.0001

Note: Model 1 adjusted for no covariates. Model 2 adjusted for age, education level, geographic region (urban or rural), annual per capita household income, survey year, and follow-up time. Model 3 additionally adjusted for physical activity, current smoking status, current alcohol drinking status, Na intake, and K intake. Model 4 additionally adjusted for BMI and WC at baseline. Model 5 additionally adjusted for SBP and DBP at baseline.

## Data Availability

The datasets generated and analyzed during the current study are available from the corresponding author (H.W.) upon reasonable request.

## Supplementary Materials

The following supporting information can be downloaded at: https://www.mdpi.com/article/10.3390/nu15030751/s1, Table S1: Parameters of model-adequacy criteria of the multi-trajectory model; Table S2: Associations between multiple trajectories with covariates and the risk of hypertension by gender; Figure S1. Mean BMI and WC in different trajectory groups in males and females at follow-up; Figure S2: Kaplan–Meier Curve for model 1 by groups for males(a) and females(b).
