# Supplementary material for: Multiple Trajectories of Body Mass Index and Waist Circumference and Their Associations with Hypertension and Blood Pressure in Chinese Adults from 1991 to 2018: A Prospective Study
Source: Nutrients. 2023 Feb 2;15(3):751. doi: 10.3390/nu15030751 (PMC9919034; doi:10.3390/nu15030751)
Supplement: Supplementary file 1 [file nutrients-15-00751-s001.zip › nutrients-2137125-supplementary.pdf]

Multiple trajectories of body mass index and waist circumference and their associations with hypertension and high blood pressure among Chinese adults from 1991 to 2018: A prospective study

Table S1. Parameters of model-adequacy criteria of the multi-trajectory model

Gender	Trajectory group number	BIC	2△BIC	AIC	OCC	APPA	Proportion of individuals in groups (%)
Male	2	-130513.12	NA	-130439.41	31.75/44.47	0.97	57.81/42.19
	3	-126821.22	7383.80	-126728.28	43.38/21.80/91.33	0.95	37.38/43.23/19.39
	4	-124807.19	4028.06	-124698.22	47.84/18.38/40.81/208.03	0.93	22.46/38.36/30.34/8.84
	5	-123955.06	1704.26	-123810.84	72.34/18.08/19.02/60.50/319.71	0.91	15.16/32.95/29.52/17.30/5.08
	6	-123401.44	1107.24	-123247.60	101.08/19.12/16.45/26.04/66.60/447.60	0.88	9.06/24.55/25.98/23.17/13.38/3.85
Female	2	-156306.78	NA	-156375.54	26.85/46.38	0.97	59.62/40.38
	3	-151766.02	9081.52	-151680.89	34.03/19.84/140.12	0.95	40.03/44.72/15.25
	4	-149681.22	4169.60	-149569.90	49.30/16.52/32.28/215.58	0.93	21.92/38.77/29.77/9.54
	5	-148698.24	1965.96	-148567.27	68.08/16.42/20.30/46.65/325.63	0.90	14.31/33.03/29.73/17.39/5.54
	6	-148091.42	1213.64	-147944.08	67.34/18.25/15.51/27.77/92.82/626.24	0.88	10.95/26.85/27.56/21.81/10.04/2.79

Note: APPA, average posterior probability of assignment. BIC, Bayesian information criterion. OCC, odds of correct classification.

Table S2. Associations between multi- trajectories with covariates and the risk of hypertension by gender

Gender	Male		Female	
Covariates	Model 5 HR and 95%CI	P	Model 5 HR and 95%CI	P
Educational level	0.91(0.86~0.97)	0.002	0.86(0.80~0.91)	<0.0001
Geographic region	0.93(0.84~1.03)	0.164	0.91(0.82~1.02)	0.094
Annual per capita household Income	1.00(1.00~1.00)	0.056	1.00(0.99~1.00)	0.271
Survey year	0.99(0.98~1.00)	0.223	0.99(0.98~1.00)	0.239
Follow-up duration	0.69(0.63~0.74)	<0.0001		
Physical activity	1.00(0.99~1.00)	0.010	1.00(0.99~1.00)	0.513
Smoking status	1.04(0.96~1.14)	0.347	0.96(0.79~1.16)	0.642
Alcohol drinking status	1.13(1.03~1.24)	0.019	0.93(0.81~1.06)	0.247
Na intake	1.00(0.99~1.00)	0.126	1.00(0.99~1.00)	0.219
K intake	1.00(0.99~1.00)	0.221	1.00(0.99~1.00)	0.285
BMI	1.02(0.99~1.04)	0.252	0.98(0.96~1.01)	0.207
WC	1.10(1.05~1.15)	0.451	1.01(1.00~1.02)	0.006
SBP	1.02(1.02~1.03)	<0.0001	1.02(1.02~1.03)	<0.0001
DBP	1.02(1.01~1.02)	<0.0001	1.01(1.00~1.02)	<0.0001

Note: Age in men analysis did not satisfy the proportional hazards assumption. Age and follow-up duration in women analysis did not satisfy the proportional hazards assumption. Their effects were not estimated here as strata variables. The covariates were continuous except for educational level (primary school and below, middle school or high school and above), geographic region (rural or urban), smoking status (non-smoker or current smoker), alcohol drinking status (non-drinker or current drinker).

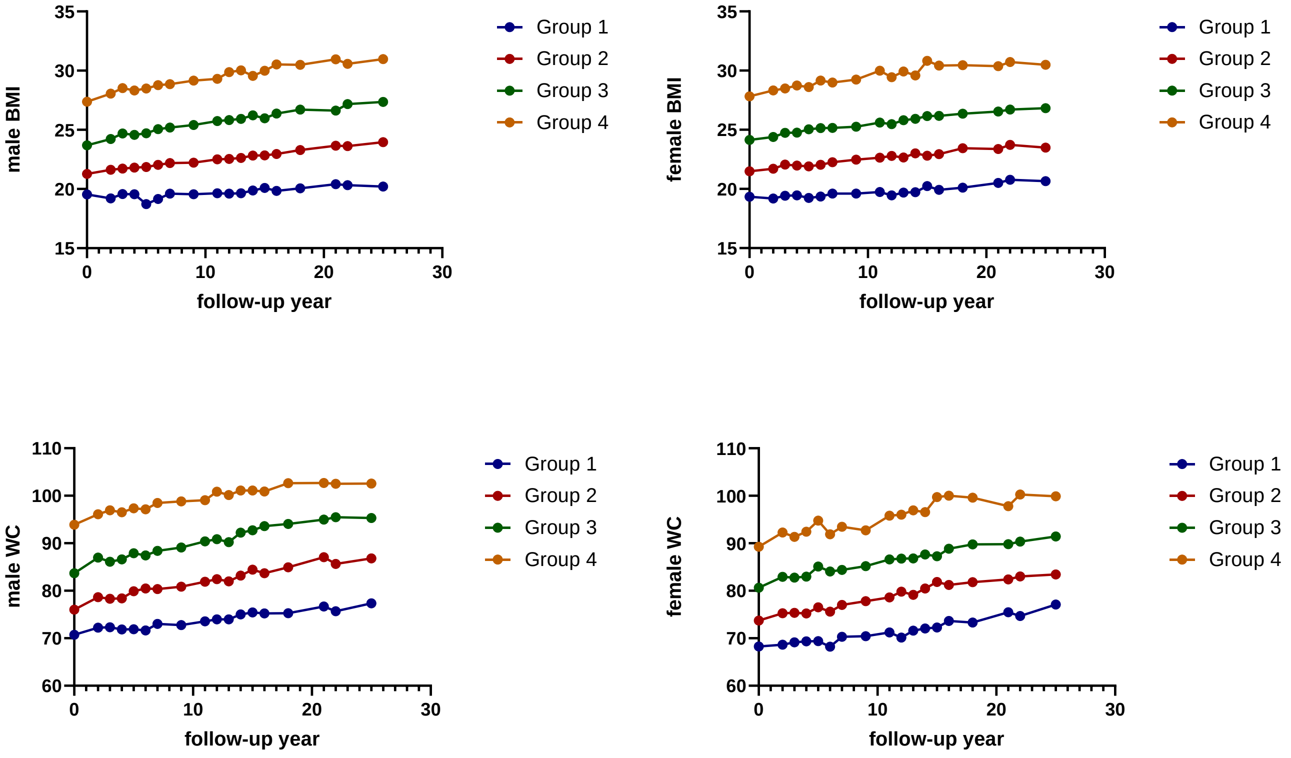

Figure S1. Mean BMI and WC in different trajectory groups in male and female at follow-up.

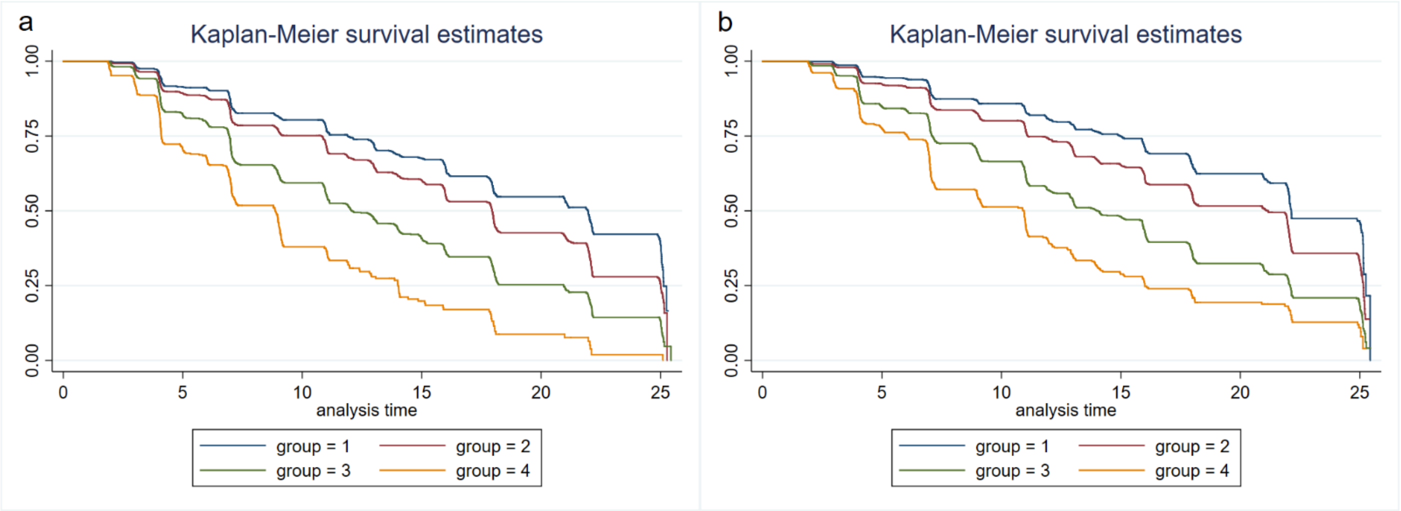

Figure S2. Kaplan-Meier Curve for model 1 by groups for males(a) and females(b).
